# Opening peer-review: the democracy of science

**DOI:** 10.1186/1477-5751-13-2

**Published:** 2014-01-24

**Authors:** Daniel R Shanahan, Bjorn R Olsen

**Affiliations:** 1 BioMed Central 6th Floor, 236 Gray’s Inn Road, London WC1X 8HB, UK; 2 Department of Cell Biology, Harvard Medical School, 240 Longwood Avenue, Boston, MA 02115, USA

## 

Scientific journals have been called the ‘minutes of science’ [1]. Born out of the exchange of letters on scientific topics and results, publication is a way of documenting what was done and, particularly in the case of open-access journals, sharing the outcome. Journal publications are considered authoritative and are generally used to inform the work of others, be it further research or, in the case of biomedicine, in treatment decisions for patients. This makes some kind of quality control all the more important.

The ‘gold standard’ for this quality control is peer review. Described as a form of self-regulation by qualified members of a profession, it was first introduced by Henry Oldenburg, the founding editor of *Philosophical Transactions of the Royal Society*, in 1665 as a means of vetting contributions to the Royal Society of London and has persisted in various forms ever since [2].

Peer review is the evaluation of a piece of work by two or more people of similar competence to the authors; but, assuming the reviewing process excludes all those involved in the direct research itself, the persons most qualified to judge the validity of a submitted research paper are precisely those who are the scientist’s closest competitors. This means that the review process can become adversarial, with referees seeming to see it as their responsibility to insist on time-consuming additions and revisions [3,4]. Moreover, under traditional, closed peer-review policies, the identity of the reviewer is withheld from the author, presenting them with a greater opportunity to act arbitrarily. It was in an effort to combat this bias that some journals introduced double-blind peer-review, whereby the author’s name was also concealed from the referee. However, research is a small world and maintaining that blinding often proved impossible.

So how did peer review, with these intrinsic issues and biases, become the judicial system of the intellectual world? Simply put, peer review is to science what democracy was to Churchill – ‘the worst form of government, except all those other forms that have been tried from time to time.’ It has served science well, with a widely-held view that, while it may not be perfect, it is nonetheless far better than anything else we have been able to devise. Indeed, the fundamental idea of peer-review seems sound; the issues lie more with the execution. Under closed systems, such as that currently enforced by the *Journal of Negative Results in BioMedicine*, there is a lack of transparency of the peer-review process and a lack of availability of evaluative information about published articles to the public. Therefore, as of February 2014, *the Journal of Negative Results in BioMedicine* will adopt an open peer-review policy.

Articles already published, or those manuscripts currently submitted, will not be affected by this change. However, for all manuscripts submitted during or after February 2014, authors will see the reviewers’ names and, if the article is published, the reading public will also see who reviewed the article and how the authors responded. This will be available as part of the pre-publication history of the published article. The peer review process will therefore be completely open and transparent, with the peer reviews being part of the record.

Research into the effect of open peer review suggests numerous benefits, in particular accountability, fairness and crediting reviewers for their efforts [5-7]. Furthermore, in a recent study, Kowalczuk *et al*. revealed that reviewer reports operating under an open peer-review system were of overall higher quality than those under a closed system, with higher scores on questions relating to feedback on the methods (11% higher), constructiveness of the comments (5% higher), and the amount of evidence provided to substantiate the comments (9% higher) [8]. Despite this, we recognise that there are also negatives. Some (junior) reviewers may feel uncomfortable signing a critical report, especially when recommending rejection [9]. This reluctance also means that more potential referees may need to be invited to review a manuscript openly than under a closed peer-review system (Parkin EC *et al.* unpublished observations) [9-11].

Reviewing an article is no easy task and many of us will have faced the situation where it feels we have put more thought into our review of the article than the authors did in designing the study and writing the manuscript. The move towards an open peer-review policy will give credit where it is due, but moreover will provide valuable information to those reading the article, sharing the referees’ critique of the manuscript and presenting all the necessary information for them to make an objective evaluation for themselves.
